# Experimental evidence and network pharmacology-based analysis reveal the molecular mechanism of Tongxinluo capsule administered in coronary heart diseases

**DOI:** 10.1042/BSR20201349

**Published:** 2020-10-13

**Authors:** Guode Li, Qingbo Xu, Kedong Han, Wenhe Yan, Chaopei Huang

**Affiliations:** Maoming People’s Hospital of Guangdong Province, No. 101 Weimin Road, Maonan District, Maoming City, Guangdong 525000, China

**Keywords:** Coronary heart disease, Molecular mechanism, Network pharmacology, TXL

## Abstract

**Background:** Tongxinluo (TXL) capsule, a polypharmacy derived from traditional Chinese medicine (TCM), has been widely used in coronary heart disease (CHD), while the underlying mechanism of TXL capsule is still unclear. The present study aimed at investigating the underlying mechanism of TXL acting on CHD patients and providing substantial evidence in molecular evidence by means of a network pharmacological analysis.

**Method:** Active compounds and targeted genes of TXL were retrieved from TCM systems pharmacology (TCMSP) and TCM integrative database (TCMID). CHD and coronary artery disease were treated as search queries in GeneCards and Online Mendelian Inheritance in Man (OMIM) databases to obtain disease-related genes. Visualization of disease–targets network was performed under administration of Cytoscape software. Besides, Gene Ontology (GO) and Kyoto Encyclopedia of Genes and Genomes (KEGG) enrichment analyses were administered. H9c2 cells were used to validate the predicted results in cardiomyocytes/reoxygenation model, and anti-inflammatory ability was examined.

**Results:** A network of a total of 212 nodes and 1016 edges was obtained. Peptide and ubiquitin-like protein ligase binding occupied a leading position of GO enrichment. For KEGG analysis, fluid shear stress and atherosclerosis, as well as inflammation-related pathways were enriched. Cellular validation revealed the anti-inflammatory effect of β-sitosterol, eriodictyol, odoricarpin, and tirucallol as active compounds of TXL.

**Conclusion:** Our study provided substantial molecular evidence that TXL capsule possessed the characteristics of multitargets with safe profile, and the main component is capable of regulating cytokine level in CHD patients.

## Introduction

Coronary heart disease (CHD), one of the most common cardiovascular diseases is caused by reduction in blood flow to cardiomyocyte owing to build-up of plaque in arteries of heart [1,2]. CHD has become a leading cause of death and the mortality increased from 5.2 million to over 7 million between 1990 and 2010 [3]. It affects individuals at any age while becomes approximately triple in progressively elder populations compared with other age groups, and the morbidity in males is larger than that in female population [4]. Statin, as the cornerstone in anti-atherosclerotic regimen, has demonstrated the substantial efficacy at reducing cardiovascular events. However, even with intensive statin therapy, many patients still suffered from high residual risks in cardiovascular events [5]. Thus, exploration of alternative anti-atherosclerotic medications with high efficacy as well as low side-effect is needed.

Traditional Chinese medicine (TCM) plays an important role in Asian population and has been popular in Western countries for its efficacy as well as less side-effects [6]. Tongxinluo (TXL) capsule, which consists of 12 principal components from plants and animal products, was approved by Food and Drug Administration (FDA) of China for treating angina pectoris and ischemic stroke [7]. Several clinical studies revealed that TXL has the ability to attenuate and stabilize atherosclerotic plaque by means of lowering serum lipid, anti-oxidation and anti-inflammation [8,9]. Furthermore, a recent multicenter randomized controlled trial, CAPITAL, demonstrated that TXL in addition to routine anti-atherosclerotic therapy could prevent the progression of intima-media thickness (IMT), plaque area and vascular remodeling [10], which provided clinic-based evidence of TXL on CHD patients. Nevertheless, the exact pharmacological effects of TXL are still unclear due to its complex formula.

**Figure 1:**
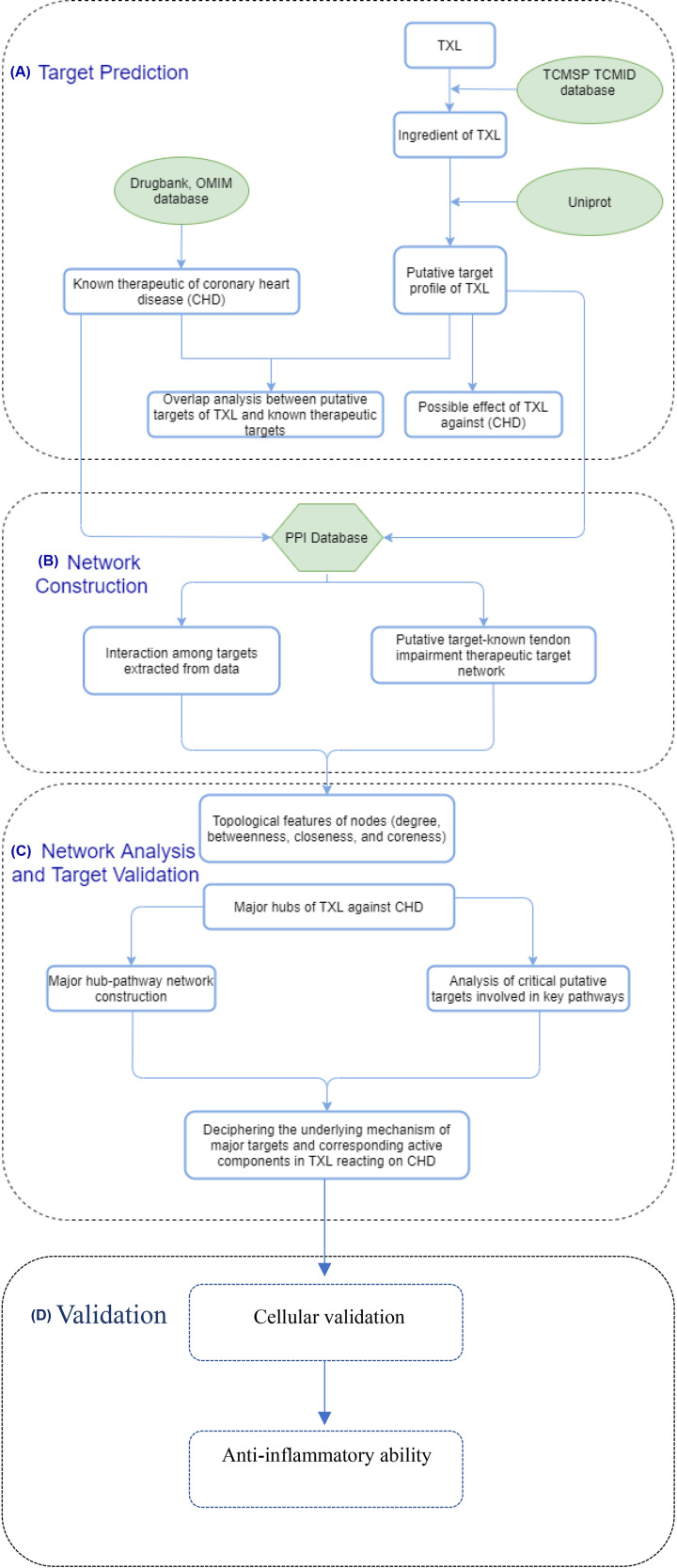
Flowchart of this network pharmacology analysis (**A**) Target prediction. (**B**) Network construction. (**C**) Network analysis and target validation. (**D**) Validation model.

With the rapid development of bioinformatics, system biology and polypharmacology, network pharmacology-based analysis has been proved to be a potent method to investigate the mechanism of TCM with complex formula [11,12]. In the present study, we aimed at investigating the mechanism of TXL exerted on CHD patients in molecular level by means of constructing a comprehensive network pharmacology-based analysis. The complete flowchart of the present study is displayed in Figure 1.

## Methods

### Chemical ingredients searching

In order to obtain the chemical ingredients of components in TXL capsule, we performed a comprehensive search on TCM systems pharmacology database (TCMSP, https://tcmspw.com/tcmsp.php) and TCM integrative database (TCMID, https://www.megabionet.org/tcmid/) by using the following queries: *Ginseng radix et rhizoma (Araliaceae; Chinese ginseng), Paeoniaeradixrubra (Paeoniaceae; Chinese peony), Ziziphispinosae semen (Rhamnaceae; jujube seed) (fried), Dalbergiaeodoriferae lignum (Dalbergia odorifera T.C.Chen; Huanghuali wood), Santalum album L. (Santalaceae; sandalwood), Olibanum (Burseraceae; Boswellia)(prepared), Borneolum (Blumea balsamifera DC.), Hirudo (Haemopidae, leech), Scorpio (Buthidae; Chinese scorpion), Scolopendra (Scolopendrasubspinipesmutilans L. Koch), Cicadae periostracum (Cicadidae; cicada), Eupolyphaga Steleophaga (Corydiidae; Woodlouse)* which are principal components of TXL [13,14]. Ingredients, molecule name, molecular weight, water partition coefficient, number of hydrogen bond donors and receptors, human oral bioavailability (OB), half-life, blood–brain barrier (BBB) and drug-likeness (DL) of each principal component were obtained from abovementioned database. Active compounds were screened out on the basis of absorption, distribution, metabolism, and excretion (ADME) protocols, with criteria of OB ≥ 30% and DL ≥ 0.18.

### Targets of active compounds

We comprehensively searched the direct targeted receptors of each active compound via DrugBank database, a specific bioinformatics and cheminformatics resource with detailed drug data, as well as targeted receptors (https://www.drugbank.ca). Full names of targeted protein receptors were obtained and converted into gene symbol on the basis of UniProt ID (https://www.uniprot.org/) for following analysis.

### Disease-related genes retrieval

GeneCards (https://www.genecards.org/) and Online Mendelian Inheritance in Man (OMIM) databases (https://www.omim.org/) were retrieved for acquiring CHD-related genes using the keywords of CHD and coronary artery disease. Intersection of retrieved targets of active compounds and disease-related genes were obtained under the administration of R (version 3.6.2) for downstream analysis.

### Visualization of ingredient-target genes-pathway and protein–protein interaction network

All intersected targets of active compounds and disease-related genes were put into Cytoscape software (Version 3.7.2) for visualization of ingredient-target genes-pathway network. To obtain interactions between intersected genes, overlapped genes were used for construction of protein–protein interaction (PPI) network in STRING database (https://string-db.org/) with the cut-off criteria of confidence > 0.4 and hiding disconnected nodes.

### Gene Ontology and Kyoto Encyclopedia of Genes and Genomes enrichment analysis

Overlapped genes were retrieved for GO and Kyoto Encyclopedia of Genes and Genomes (KEGG) enrichment analysis with the criterion of *P*-value <0.05. Bar plots of GO and KEGG were exported and signal pathways involved in this network analysis were visualized in forms of diagram.

### Reagents used in validation

β-sitosterol, ellagic acid, formononetin, eriodictyol, were purchased from MedChemExpress (MCE, Shanghai, China) with the purity > 98%. Odoricarpin was purchased from TASLY PHARM (Tianjing, China) with the purity > 98%. Tirucallol was purchased from Shanghai Institute of Biotechnology Co., Ltd. (Shanghai, China) with the purity > 98%.

### Cells

H9c2 cells were purchased from Tongpai Technology Company (Shanghai, China) and cultured in Dulbecco’s modified Eagle’s medium (DMEM) bought from Thermo Fisher Scientific (Guangzhou, China), with the supplement of 10% v/v FBS and 1% v/v penicillin/streptomycin in CO_2_ incubator at 37°C and 95% relative humidity.

### Cell models

Regarding the investigation of the protective effect of TXL, hypoxia/reoxygenation (H/R) model was administered. Cells were put into an incubator with Krebs–Ringer bicarbonate buffer medium saturated with 99.99% N_2_ for 140 min [15]. Cells were reoxygenated through changing the DMEM back and cultured under normal oxygen level (21%) for 1 h. The molecules were applied for 48 h before hypoxia until the end of oxygenation.

### Cell viability test

Cell viability test was performed under the assistance of cell counting kit-8 (CCK-8) after the administration of abovementioned active components of TXL. Cells with different molecules were seeded in a 96-well plate at a density of 1 × 10^4^ cells for 24 h. Then, 10% CCK-8 was added and OD value was read at 450 nm after 1 h. In addition, optimal concentration of each molecule was explored ranging from 5 to 100 μM [16–19]. Each cell viability test with different molecules was repeated five times and measurement of relative cell viability was recorded.

### Investigation of anti-inflammatory effect

For anti-inflammatory effect, cells were seeded in a 96-well plate incubated for 24 h, and treated with 0.01 μg/ml LPS 30 min after incubation with optimal concentration of abovementioned molecules was obtained. Then, supernatant was collected by adding 150 μl dimethyl sulfoxide (DMSO) and stored at −80°C for downstream analysis. Concentration of cytokine was measured by enzyme-linked immunosorbent assay (ELISA) under corresponding protocol and IL-6 (K4144-100, Biovision) and IL-8 (K4169-100, Biovision) ELISA kits were administered in the presentstudy. Each test with different molecules was repeated five times and average concentration of corresponding results was recorded.

## Results

### Identification of putative ingredient targets

With the mentioned search queries of *Panax Ginseng C. A. Mey., Radix Paeoniae Rubra, Ziziphi Spinosae Semen, Dalbergiae Odoriferae lignum, Santalum Album L., Olibanun, Cicadae Periostracum, Borneolum Syntheticum, hirudo, Scorpio, Scolopendra, Cicadae periostracum* and criteria of OB ≥ 30% as well as DL ≥ 0.18, a total of 111 chemical ingredients were collected within TXL prescription from TCMSP and TCMID databases. Besides, the targeted genes of each retrieved chemical ingredients were explored and a total of 1205 targeted genes were obtained. The names of targeted genes were converted into gene ID on basis of UniProt database, and eventually 861 eligible targeted genes with molecular names and symbol ID were acquired. The active compounds involved in the present study with the amount as well as ratio of each component [20] were shown in Table 1 and detailed information of putative ingredients with targeted genes were documented in Supplementary Table S1.

**Table 1:** Detailed information of active ingredients of TXL

Local name	Latin scientific names	Mol ID	Molecule name	Ratio*	MW	AlogP	OB (%)	DL
Ginseng radix et rhizoma	Panax Ginseng C. A. Mey			0.024 (9.4)				
		MOL002879	Diop		390.62	7.44	43.59	0.39
		MOL000449	Stigmasterol		412.77	7.64	43.83	0.76
		MOL000358	Beta-sitosterol		414.79	8.08	36.91	0.75
		MOL003648	Inermin		284.28	2.44	65.83	0.54
		MOL000422	Kaempferol		286.25	1.77	41.88	0.24
		MOL004492	Chrysanthemaxanthin		584.96	8.24	38.72	0.58
		MOL005308	Aposiopolamine		271.34	1.39	66.65	0.22
		MOL005314	Celabenzine		379.55	2.29	101.88	0.49
		MOL005317	Deoxyharringtonine		515.66	3.13	39.27	0.81
		MOL005318	Dianthramine		289.26	2.05	40.45	0.2
		MOL005320	Arachidonate		304.52	6.41	45.57	0.2
		MOL005321	Frutinone A		264.24	2.7	65.9	0.34
		MOL005344	Ginsenoside rh2		622.98	4.04	36.32	0.56
		MOL005348	Ginsenoside-Rh4_qt		458.8	5.59	31.11	0.78
		MOL005356	Girinimbin		263.36	4.6	61.22	0.31
		MOL005357	Gomisin B		514.62	2.73	31.99	0.83
		MOL005360	Malkangunin		432.56	1.84	57.71	0.63
		MOL005376	Panaxadiol		460.82	5.46	33.09	0.79
		MOL005384	Suchilactone		368.41	3.73	57.52	0.56
		MOL005399	Alexandrin_qt		414.79	8.08	36.91	0.75
		MOL005401	Ginsenoside Rg5_qt		442.8	6.8	39.56	0.79
		MOL000787	Fumarine		353.4	2.95	59.26	0.83
Paeoniaeradixrubra	Paeoniaceae			0.022 (8.6)				
		MOL001918	Paeoniflorgenone		318.35	0.79	87.59	0.37
		MOL001925	Paeoniflorin_qt		318.35	0.46	68.18	0.4
		MOL007016	Paeoniflorigenone		318.35	0.79	65.33	0.37
		MOL006996	1-o-beta-d-glucopyranosylpaeonisuffrone_qt		332.38	0.51	65.08	0.35
		MOL007022	EvofolinB		318.35	2.07	64.74	0.22
		MOL007018	9-ethyl-neo-paeoniaflorin A_qt		334.4	1.48	64.42	0.3
		MOL006992	(2R,3R)-4-methoxyl-distylin		318.3	1.89	59.98	0.3
		MOL007008	4-ethyl-paeoniflorin_qt		332.38	1.02	56.87	0.44
		MOL007012	4-o-methyl-paeoniflorin_qt		332.38	0.87	56.7	0.43
		MOL000492	(+)-catechin		290.29	1.92	54.83	0.24
		MOL001924	Paeoniflorin		480.51	−1.28	53.87	0.79
		MOL001921	Lactiflorin		462.49	−0.57	49.12	0.8
		MOL007005	Albiflorin_qt		318.35	0.42	48.7	0.33
		MOL000449	Stigmasterol		412.77	7.64	43.83	0.76
		MOL001002	Ellagic acid		302.2	1.48	43.06	0.43
		MOL004355	Spinasterol		412.77	7.64	42.98	0.76
		MOL002776	Baicalin		446.39	0.64	40.12	0.75
		MOL005043	Campest-5-en-3beta-ol		400.76	7.63	37.58	0.71
		MOL006999	Stigmast-7-en-3-ol		414.79	8.08	37.42	0.75
		MOL000358	Beta-sitosterol		414.79	8.08	36.91	0.75
		MOL000359	Sitosterol		414.79	8.08	36.91	0.75
		MOL006994	1-o-beta-d-glucopyranosyl-8-o -benzoylpaeonisuffrone_qt		302.35	0.44	36.01	0.3
		MOL002714	Baicalein		270.25	2.33	33.52	0.21
		MOL002883	Ethyl oleate (NF)		310.58	7.44	32.4	0.19
		MOL007014	8-debenzoylpaeonidanin		390.43	-3.28	31.74	0.45
		MOL007003	Bnzoyl paeoniflorin		584.62	0.76	31.14	0.54
		MOL007025	Isobenzoylpaeoniflorin		584.62	0.76	31.14	0.54
		MOL006990	(1S,2S,4R)-trans-2-hydroxy-1,8-cineole-B-d-glucopyranoside		332.44	−0.57	30.25	0.27
		MOL007004	Albiflorin		480.51	−1.33	30.25	0.77
Ziziphispinosae semen	Rhamnaceae			0.024 (9.4)				
		MOL001522	(S)-Coclaurine		285.37	2.83	42.35	0.24
		MOL001546	Zizyphusine		342.45	3.12	41.53	0.55
		MOL001527	Jujuboside A_qt		472.78	4.39	34.96	0.62
		MOL001542	Swertisin		446.44	0.19	31.83	0.75
		MOL001525	Daucosterol		414.79	8.08	36.91	0.75
		MOL001532	Phytosterol		414.79	8.08	36.91	0.75
		MOL001521	Ceanothic acid		486.76	5.36	33.41	0.77
		MOL000211	Mairin		456.78	6.52	55.38	0.78
		MOL001539	Sanjoinenine		489.67	4.23	67.28	0.79
Dalbergiaeodoriferae lignum	Dalbergia odorifera T.C.Chen			0.020 (7.8)				
		MOL002958	3′-Hydroxymelanettin		300.28	2.56	30.69	0.27
		MOL001792	DFV		256.27	2.57	32.76	0.18
		MOL002957	9-O-Methylcoumestrol		282.26	3.26	33.73	0.38
		MOL002982	(3R,4R)-3′,7-dihydroxy-2′,4′-dimethoxy-4-[(2S)-4′,5,7-trihydroxyflavanone-6-yl]isoflavan		572.6	4.88	33.96	0.63
		MOL002967	7-hydroxy-4′-methoxy-2′,5′-dioxo-4-[(3R)-2′,7-dihydroxy-4′-methoxyisoflavan-5′-yl]isoflavane		556.6	4.26	34.78	0.7
		MOL003000	Stevein		284.28	2.83	36.54	0.24
		MOL000359	Sitosterol		414.79	8.08	36.91	0.75
		MOL000358	β-sitosterol		414.79	8.08	36.91	0.75
		MOL002991	(6aR,11aR)-3,9-dimethoxy-6a,11a-dihydro -6H-benzofurano[3,2-c]chromene-4,10-diol		316.33	2.37	38.96	0.48
		MOL002963	4′,5′,7-trimethyl-3-methoxyflavone		294.37	4.1	40.66	0.25
		MOL002914	Eriodyctiol (flavanone)		288.27	2.03	41.35	0.24
		MOL001040	(2R)-5,7-dihydroxy-2-(4-hydroxyphenyl) chroman-4-one		272.27	2.3	42.36	0.21
		MOL002962	(3S)-7-hydroxy-3-(2,3,4-trimethoxyphenyl) chroman-4-one		330.36	2.67	48.23	0.33
		MOL002989	4-Hydroxyhomopterocarpin		300.33	2.64	48.41	0.43
		MOL002959	3′-Methoxydaidzein		284.28	2.32	48.57	0.24
		MOL002565	Medicarpin		270.3	2.66	49.22	0.34
		MOL002940	(3R)-3-(2,3-dihydroxy-4-methoxyphenyl) -7-hydroxychroman-4-one		302.3	2.16	52.06	0.27
		MOL003001	Vestitone		286.3	2.43	52.83	0.24
		MOL002996	Odoricarpin		330.36	2.63	55.02	0.53
		MOL000228	(2R)-7-hydroxy-5-methoxy-2- phenylchroman-4-one		270.3	2.82	55.23	0.2
		MOL002973	Bowdichione		298.26	0.64	55.78	0.28
		MOL000380	(6aR,11aR)-9,10-dimethoxy-6a,11a-dihydro- 6H-benzofurano[3,2-c]chromen-3-ol		300.33	2.64	64.26	0.42
		MOL002990	(6aR,11aR)-3,9,10-trimethoxy-6a,11a-dihydro-6H-benzofurano[3,2-c]chromen-4-ol		330.36	2.63	66.86	0.53
		MOL002938	(3R)-4′-Methoxy-2′,3, 7-trihydroxyisoflavanone		302.3	1.83	68.86	0.27
		MOL002950	(3R)-7,2′,3′-trihydroxy-4 -methoxyisoflavan		288.32	2.21	69.65	0.24
		MOL000392	Formononetin		268.28	2.58	69.67	0.21
		MOL002975	Butin		272.27	2.3	69.94	0.21
		MOL002961	(-)-Vestitol		272.32	3.15	70.29	0.21
		MOL002981	Duartin		332.38	3.11	70.63	0.34
		MOL003003	Xenognosin B		284.28	2.32	72.71	0.24
		MOL002985	Isoduartin		332.38	3.11	74.11	0.34
		MOL002966	Dalbergin		268.28	3.1	78.18	0.2
		MOL003002	Violanone		316.33	2.42	80.24	0.3
		MOL002941	(3R)-3-(2,3-dihydroxy-4-methoxyphenyl) chroman-7,8-diol		304.32	2.61	82.35	0.27
		MOL002939	(3R)-5′-Methoxyvestitol		302.35	3.13	83.06	0.26
		MOL002999	Sativanone		300.33	2.68	85.63	0.27
		MOL002997	3-(2-hydroxy-3,4-dimethoxyphenyl)-2H-chromen-7-ol		300.33	2.95	86.18	0.27
Santalaceae	Santalum album L.			0.008 (3.1)				
		MOL000354	Isorhamnetin		316.28	1.76	49.6	0.31
		MOL000006	luteolin		286.25	2.07	36.16	0.25
		MOL002322	Isovitexin		432.41	−0.06	31.29	0.72
Olibanum	Burseraceae			0.008 (3.1)				
		MOL001215	Tirucallol		426.8	8.12	42.12	0.75
		MOL001241	O-acetyl-α-boswellic acid		498.82	6.8	42.73	0.7
		MOL001243	3alpha-Hydroxy-olean-12-en- 24-oic-acid		456.78	6.42	39.32	0.75
		MOL001255	Boswellic acid		456.78	6.47	39.55	0.75
		MOL001263	3-oxo-tirucallic,acid		454.76	6.99	42.86	0.81
		MOL001265	Acetyl-alpha-boswellic,acid		498.82	6.8	42.73	0.7
		MOL001272	Incensole		306.54	4.97	45.59	0.22
		MOL001295	Phyllocladene		272.52	5.63	33.4	0.27
Borneolum	Blumea balsamifera DC.			0.006 (2.3)				
		MOL006862	Bronyl acetate		447.55	4.02	59.3	0.51
		MOL006861	Asiatic acid		488.78	4.3	41.38	0.71
		MOL006865	Dipterocarpol		442.8	6.92	41.71	0.76
Cicadidae	Cicadae Periostracum			0.031 (11.7)				
Hirudo	Haemopidae			0.049 (18.8)				
Scorpio	Buthidae			0.031 (11.7)				
Scolopendra	Scolopendrasubspinipesmutilans L. Koch			0.006 (2.4)				
EupolyphagaSteleophaga	Corydiidae			0.031 (11.7)				

* Ratio is displayed in form of g (%), and one tablet of TXL is 0.26 g.

Abbreviations: AlogP, partition coefficient of concentration of drug in octanol/concentration of drug in aqueous solution; MW, molecular weight.

### Identification of disease-related genes

Since the application of TXL is to lower serum lipid level, anti-oxidation and anti-inflammation, which are standard management in CHD [21], the CHD and coronary artery disease were treated as keywords to acquire relevant genes. After the administration of search queries in GeneCards and OMIM databases, a total of 7389 CHD-relevant genes were obtained. Furthermore, intersection between ingredients-targeted and CHD-relevant genes were performed and 138 overlapped genes were obtained eventually. The Venn diagram of overlapped genes were displayed in Supplementary Figure S1.

### Network visualization

**Figure 2:**
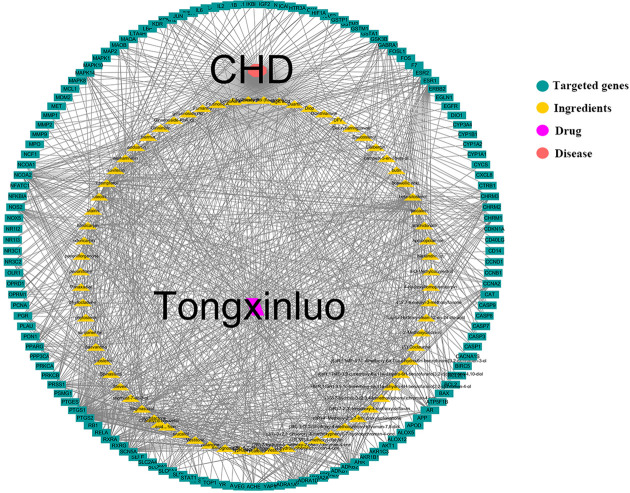
Ingredient–target network

A complete ingredient–target network consisting of a total of 212 nodes and 1016 edges (138 target nodes, 72 putative ingredients nodes, 1 disease node and 1 TXL node) was obtained after administration of Cytoscape software as shown in Figure 2. For detailed information, each node included in this ingredient–target network was documented in Supplementary Table S2.

**Figure 3:**
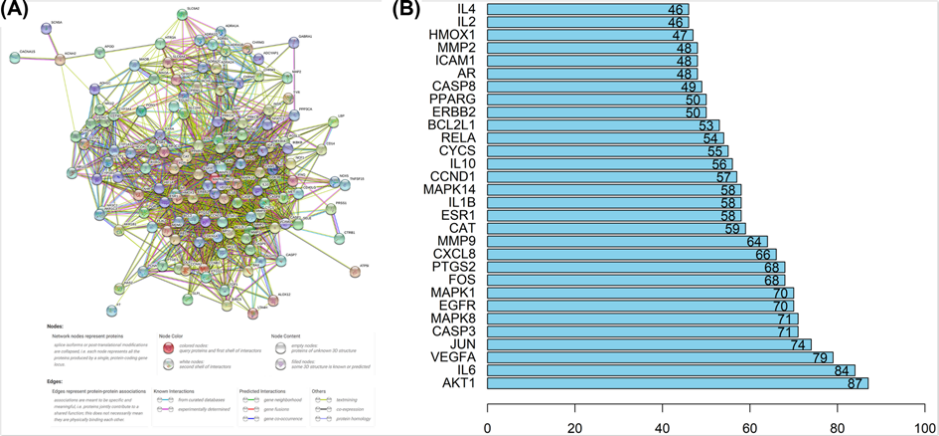
Overlapped genes interaction (**A**) PPI network showing interactions between the involved genes. (**B**) Frequency of targets within PPI network.

Overlapped genes were processed by STRING to produce a PPI network with confidence > 0.4 and shown in Figure 3A. PPIs were displayed by a total of 138 nodes and 1939 edges with average node degree of 28.1. Within the PPI network, AKT1 showed high degree in coreness of 87-times interaction, followed by IL6 (84 times), VEGFA (79-times), JUN (74-times), CASP3 (71-times), MAPK8 (71-times), respectively. Top 30 proteins with highest interaction time are shown in Figure 3B and the detailed information of PPI is documented in Suplementary Table S3.

### GO and KEGG enrichment analyses

**Figure 4:**
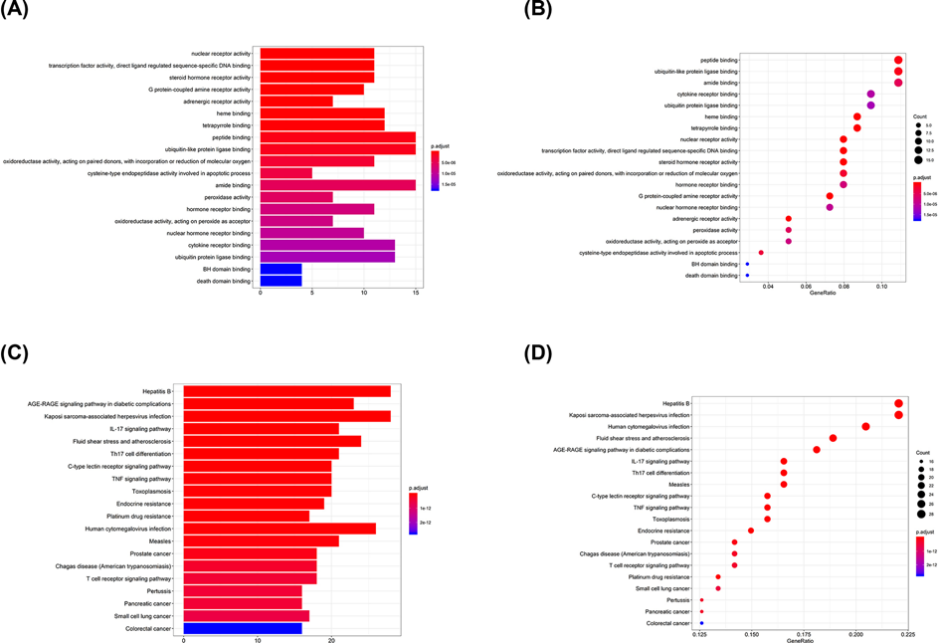
GO and KEGG enrichment analyses (**A**) Box plot of GO enrichment. (**B**) Dot plot of GO enrichment. (**C**) Box plot of KEGG enrichment. (**D**) Dot plot of KEGG enrichment.

Overlapped genes’ names were converted into symbol ID via UniProt database for GO and KEGG enrichment analyses. Regarding GO enrichment analysis, function of peptide binding and ubiquitin-like protein ligase binding occupied the leading position among all relevant genes with adjusted *P*-value of 6.35e^−7^ and 1.00e^−6^, respectively. Heme binding and tetrapyrrole binding function were at second place of overlapped genes enrichment analysis with adjusted *P*-value of 3.49e^−8^ and 6.83e^−8^, respectively. Top 20 categories of GO enrichment analysis are shown in Figure 4A,B.

When it comes to KEGG enrichment analysis, AGE-RAGE signaling pathway, and fluid shear stress and atherosclerosis pathway occupied the predominant position with adjusted *P*-value of 5.60e^−19^ and 3.88e^−17^, respectively. Moreover, inflammation-related pathways, such as IL-17, TNF and T-cell receptors signaling pathways, were principal pathways within the TXL-CHD overlapped genes enrichment, with the adjusted *P*-value of 3.19e^−17^, 1.13e^−14^, 3.73e-13, respectively. Top 20 categories of KEGG enrichment analysis are shown in Figure 4C,D. Furthermore, Pathviews of fluid shear stress and atherosclerosis, IL-6, TNF, toll-like receptor, and T-cell receptor signaling pathways are displayed in Supplementary Figure S2.

### Active ingredients protect H9c2 cells from H/R injury

**Figure 5:**
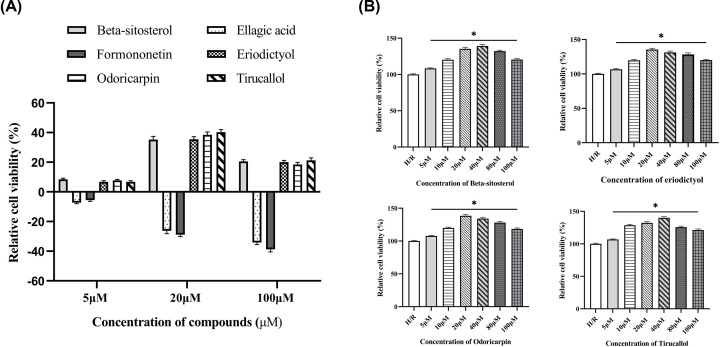
Cell viability test of active compounds of TXL (**A**) Different concentrations of active compounds on improvement rate (*n*=5). (**B**) Exploration of optimal dosage of active compounds on improvement rate (*n*=5). * indicates existence of significance (*P*<0.05).

Six potential ingredients, β-sitosterol, ellagic acid, formononetin, eriodictyol, odoricarpin, tirucallol (detailed information shown in Table 2), were obtained and used for validation. Regarding to cell viability tests, β-sitosterol, eriodictyol, odoricarpin and tirucallol revealed positive improvement effect, while ellagic acid and formononetin were found to be cytotoxic to H9c2 cells in H/R model (Figure 5A). Improvement rate at different concentrations was investigated to obtain optimal dosage. From the results, the optimal dosage of β-sitosterol, eriodictyol, odoricarpin, tirucallol were 40, 20, 20 and 40 μM in this model, respectively, and decreased relative cell viability was observed in each test when concentration exceeded 50 μM (Figure 5B).

**Table 2:** Molecules used in validation

Mole ID	Molecule name	MW	OB (%)	DL	Structure
MOL000358	β-sitosterol	414.79	36.91	0.75	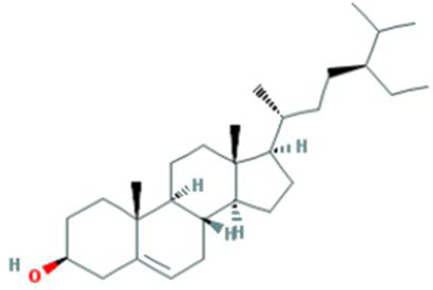
MOL001002	Ellagic acid	302.2	43.06	0.43	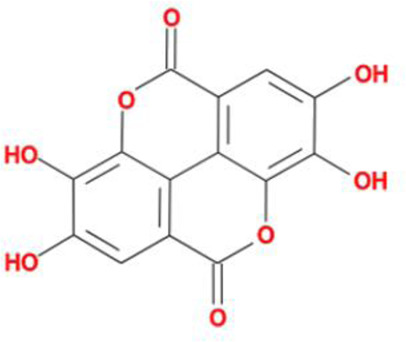
MOL000392	Formononetin	268.28	69.67	0.21	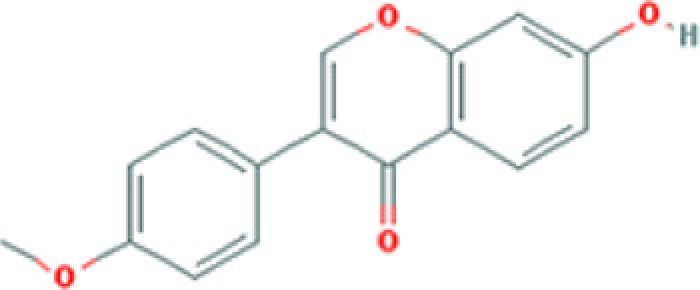
MOL002914	Eriodictyol	288.27	41.35	0.24	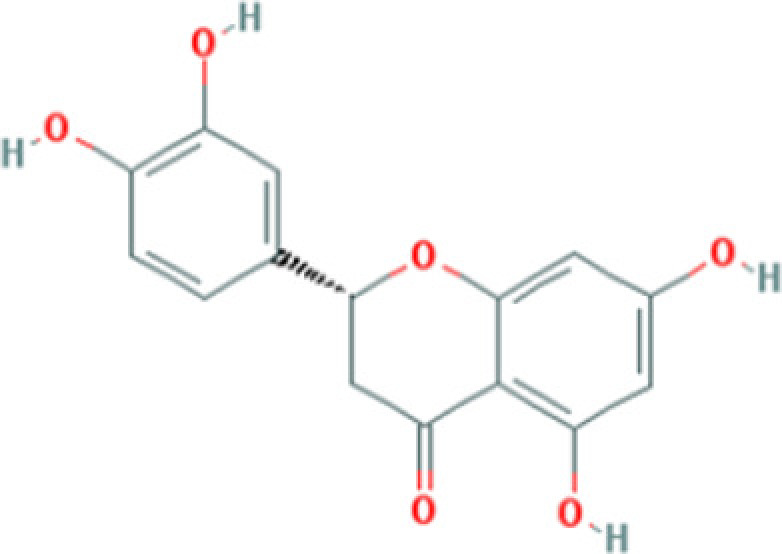
MOL002996	Odoricarpin	330.36	55.02	0.53	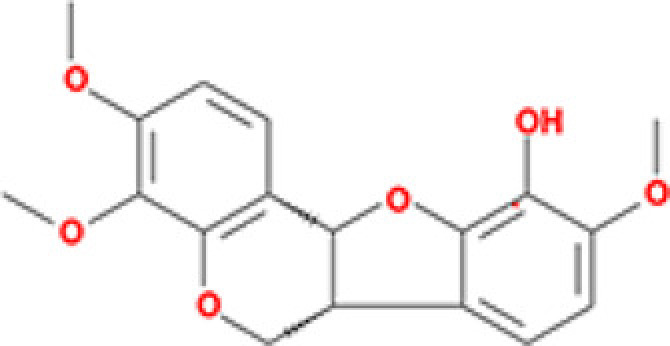
MOL001215	Tirucallol	426.8	42.12	0.75	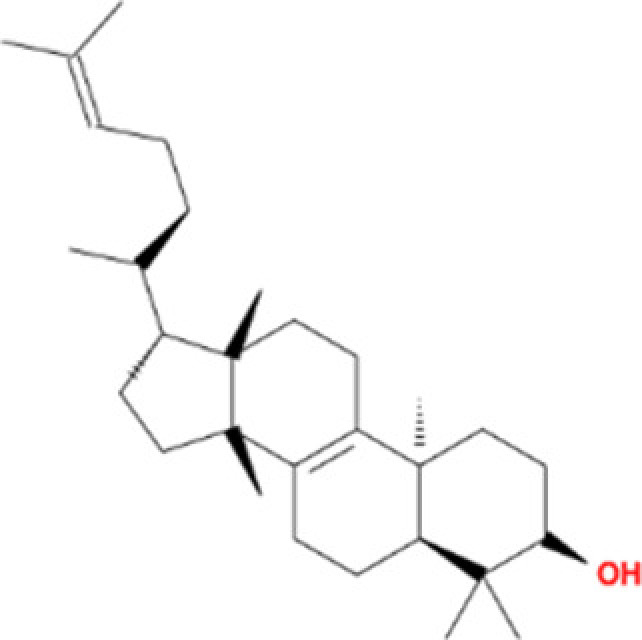

Abbreviation: MW, molecular weight.

### Anti-inflammatory effect of TXL

**Figure 6:**
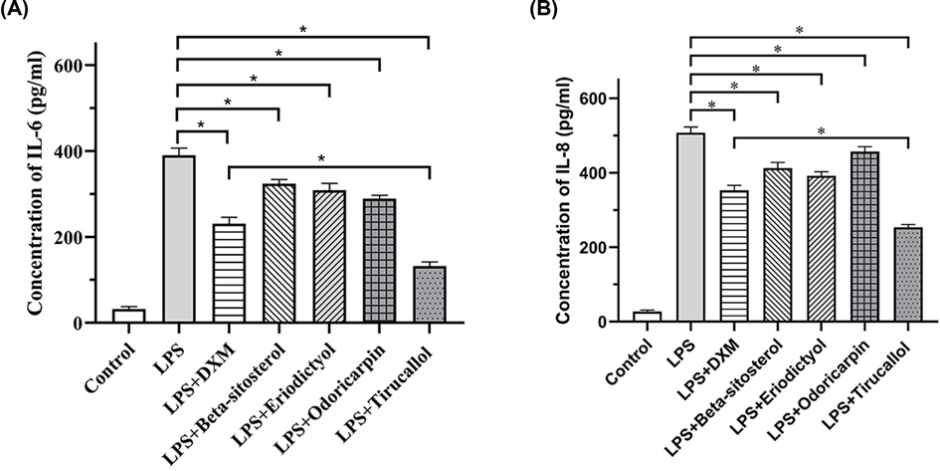
Anti-inflammatory ability of active compounds (**A**) Effects of active compounds on IL-6 concentration (*n*=5). (**B**) Effects of active compounds on IL-8 concentration (*n*=5). * indicates existence of significance (*P*<0.05).

Due to the significance of anti-inflammatory regulation in CHD management, the anti-inflammatory effect of TXL was investigated. Since the enriched pathways in anti-inflammatory regulation (Supplementary Figure S2), concentrations of IL-6 (Figure 6A) and IL-8 (Figure 6B) were investigated with the abovementioned optimal concentration of four compounds. β-sitosterol, eridictyol, odoricarpin and tirucallol indicated significant inhibition on concentration of IL-6 as well as IL-8 (*P*<0.05). Moreover, tirucallol revealed to have a significant anti-inflammation effect compared with DXM group (*P*<0.05). Collectively, active compounds of TXL is capable of regulating anti-inflammation.

## Discussion

In previous study, resistance to statin regimen led to rapid progression of atheroma, indicating warranted alternative to lipid-lowering medication [22]. As indicators of plaque progression, IMT and maximal plaque area are favored indicators for CHD assessment. In CAPITAL trial, as the additional anti-atherosclerotic regimen to routine CHD therapy, TXL revealed superiority compared with control group in slowing down the progression of CHD significantly [10]. However, the underlying anti-atherosclerotic effects of TXL were unclear. After this research, substantial evidences might be provided at the molecular level.

Network pharmacology was designed for investigating single-medication targeting on multiple targets so as to enhance efficacy as well as reducing toxicity to patients [23]. Besides, TXL capsule was a mixture of 12 plant and animal products with multiple ingredients and targets, which conformed to the abovementioned perspective and was proved to be effective in cellular level in the present study.

Regarding enrichment analysis, several pathways revealed the potential mechanism of TXL capsule acting on anti-atherosclerotic events. Peptide and ubiquitin-like protein ligase binding occupied the predominant position among GO enrichment analysis, in which rising ubiquitin was reported as positively correlated indicators with the severity of pathologies such as trauma, burn, and especially in CHD and acute myocardial infarction (AMI) patients [24–26]. Also, extracellular ubiquitin was shown to be elevated in CHD patients, especially in patients with acute coronary syndrome (ACS) attack, and it was positively related to Gensini score reflecting the degree of atherosclerosis in CHD [27]. Moreover, ubiquitin was suggested to be positively related to inflammatory markers CRP, CK-MB and cTnl, which were associated with progression of atherosclerosis as well as AMI [28]. To sum up, ubiquitin is an alternative biomarker to predict the severity of CHD. Predominant function of targeted genes on ubiquitin-like protein ligase binding might hint that TXL capsule had the capacity on regulating extracellular ubiquitin level to prevent the progression of atherosclerosis.

Fluid shear stress and atherosclerosis pathway was enriched in KEGG analysis, and it was found to be associated with microvascular and epicardial endothelial dysfunction in CHD patients. Coronary arteries exposed to abnormal microvascular endothelial function exhibited significantly lower shear stress compared with normal coronary arteries [29]. Apart from systemic risk factors, local factors as low shear stress might contribute to promotion of early focal epicardial endothelial dysfunction and potential plaque progression [30,31]. A fall in shear stress might be triggered by microvascular endothelial dysfunction which induced by established systemic risk factors like inflammation and oxidative stress at early stage of disease, further provoking as well as exacerbating inflammatory processes of coronary endothelium. Moreover, inflammation plays an indispensable role in the progression of atherosclerosis [32,33], and inflammation-related pathways such as IL-17, TNF, toll-like receptor, T-cell receptor signaling pathways, were enriched among KEGG analysis. Targeted anti-inflammatory regimen and reduction in CRP have been shown to reduce major adverse cardiovascular events in established CHD patients [34,35]. As discussed above, TXL was also capable of regulating ubiquitin to adjust CRP level, and the active compounds of TXL were validated to be effective in regulating inflammation-related pathway, which further confirmed the theory of anti-inflammatory effects of TXL capsule on CHD patients.

However, several limitations should be considered in the present study. First, retrieved active ingredients might be inconsistent with the exact compounds absorbed by patients. Second, only targeted genes of active ingredients could be found but the exploration of predominantly targeted genes by active compounds is difficult. Third, errors might occur in GO and KEGG enrichment analyses due to the complex formula of TXL capsule and enriched pathway might be confused. Last but not the least, validation is performed at cellular level and the verification in animal model to investigate more indicators is still necessary in future research.

## Conclusion

Our study provided substantial molecular evidence that TXL capsule possessed the characteristics of multitargets with safe profile, and its main component is effective in regulating cytokine level as well as improving hypoxia to protect myocardial cells on CHD patients.

## Supplementary Material

Supplementary Figures S1-S2

Supplementary Tables S1-S3

## Abbreviations

AMI: acute myocardial infarction
CCK-8: cell counting kit-8
CHD: coronary heart disease
CK-MB: creatine kinase-MB
CRP: C-reactive protein
cTn1: cardiac troponin 1
DL: drug-likeness
DMEM: Dulbecco’s modified Eagle’s medium
ELISA: enzyme-linked immunosorbent assay
GO: gene ontology
H/R: hypoxia/reoxygenation
IMT: intima-media thickness
KEGG: Kyoto Encyclopedia of Genes and Genomes
LPS: lipopolysaccharide
OB: oral bioavailability
OMIM: Online Mendelian Inheritance in Man
PPI: protein–protein interaction
TCM: Traditional Chinese medicine
TCMID: TCM integrative database
TCMSP: TCM systems pharmacology
TXL: tongxinluo
