# Supplementary material for: Experimental evidence and network pharmacology-based analysis reveal the molecular mechanism of Tongxinluo capsule administered in coronary heart diseases
Source: Biosci Rep. 2020 Oct 13;40(10):BSR20201349. doi: 10.1042/BSR20201349 (PMC7560518; doi:10.1042/BSR20201349)
Supplement: Supplementary Figures S1-S2 [file BSR-2020-1349_supp.pdf]

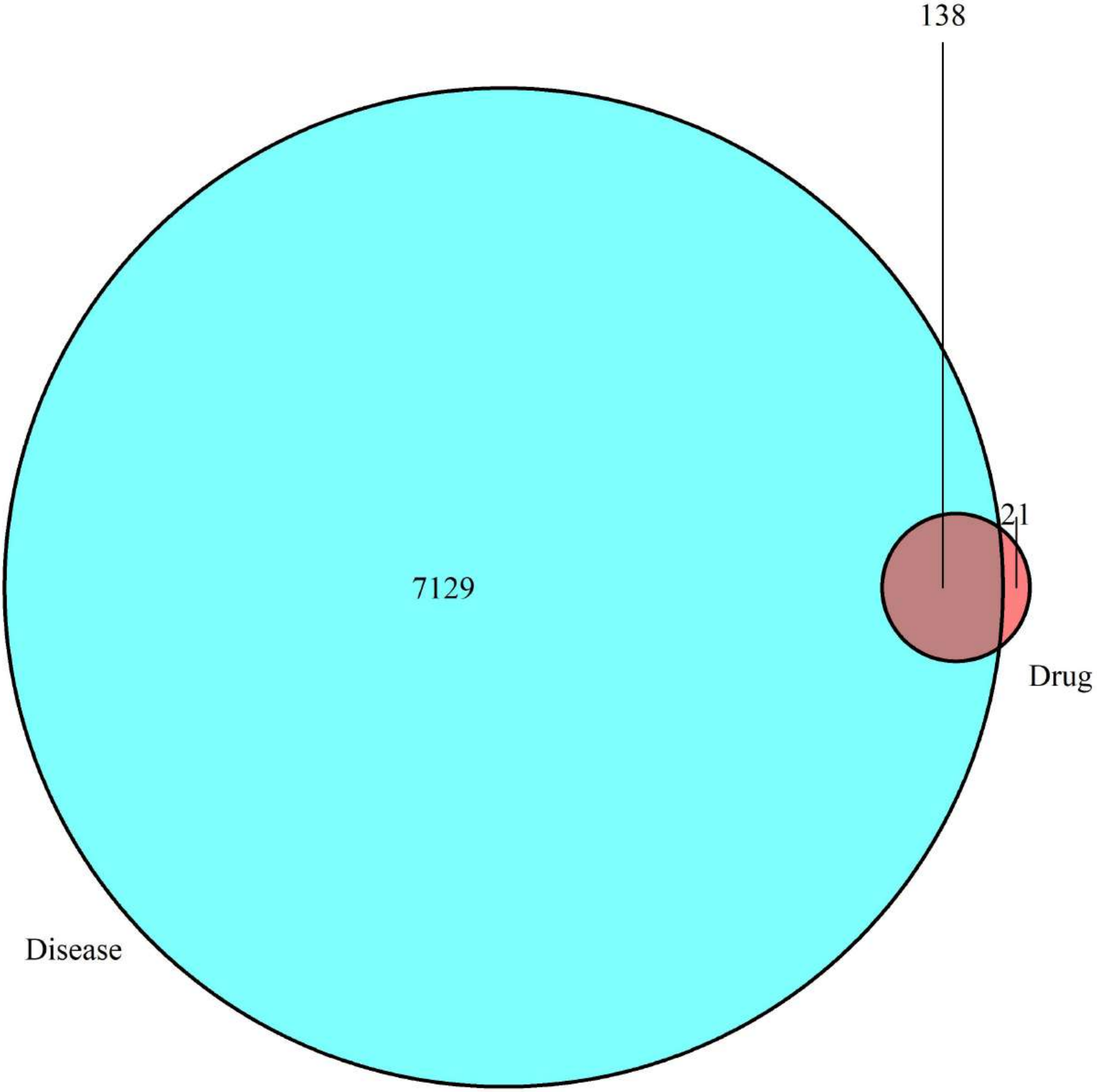

138
7129
21
Drug
Disease

Figure S1. Venn diagram showing the overlapped genes between TXL and CHD.

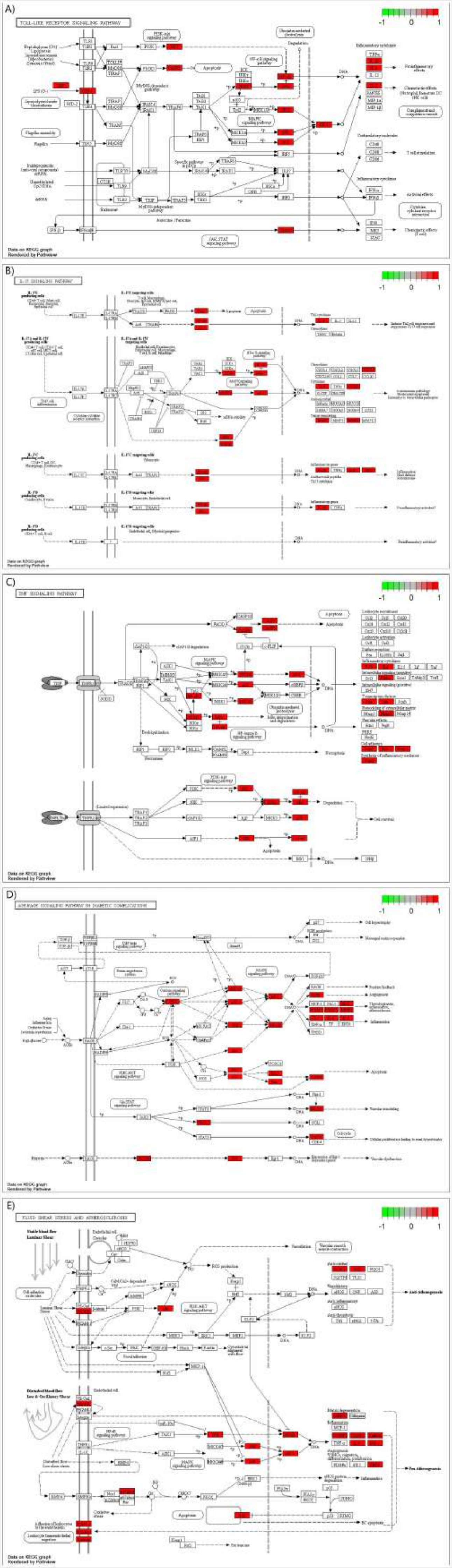

A)
TOLL-LIKE RECEPTOR SIGNALING PATHWAY
-1 0 1
Data on KEGG graph
Rendered by Pathview
B)
IL-17 SIGNALING PATHWAY
-1 0 1
Data on KEGG graph
Rendered by Pathview
C)
TNF SIGNALING PATHWAY
-1 0 1
Data on KEGG graph
Rendered by Pathview
D)
AGE-RAGE SIGNALING PATHWAY IN DIABETIC COMPLICATIONS
-1 0 1
Data on KEGG graph
Rendered by Pathview
E)
FLUID SHEAR STRESS AND ATHEROSCLEROSIS
-1 0 1
Data on KEGG graph
Rendered by Pathview

Figure S2. Pathviews of involved pathways. A) Pathviews of Toll-like receptor signaling pathway. B) Pathviews of IL-17 signaling pathway. C) Pathviews of TNF signaling pathway. D) Pathviews of AGE-RAGE signaling pathway. E) Pathviews of Fluid shear stress and Atherosclerosis.
